# Visual improvement and regeneration of retinal layers in eyes with small, medium, and large idiopathic full-thickness macular holes treated with the inverted internal limiting membrane flap technique over a period of 12 months

**DOI:** 10.1007/s00417-022-05676-9

**Published:** 2022-04-27

**Authors:** Nathalie Bleidißel, Julia Friedrich, Nikolaus Feucht, Julian Klaas, Mathias Maier

**Affiliations:** 1 Department of Ophthalmology, Klinikum rechts der Isar, Technical University Munich (TUM), Ismaningerstraße 22, 81675 Munich, Germany; 2 Smile Eyes Augenklinik Airport, Terminalstraße Mitte 18, 85356 Munich, Germany; 3 Department of Ophthalmology, University Hospital Munich (LMU), Mathildenstraße 8, 80336 Munich, Germany

**Keywords:** Inverted internal limiting membrane flap technique, Macular hole size, Spectral‐domain optical coherence tomography, External limiting membrane, Ellipsoid zone, Defect length

## Abstract

**Purpose:**

This study aims to compare the improvement of best-corrected visual acuity (BCVA) and the reduction in defect length of external limiting membrane (ELM) and ellipsoid zone (EZ) in small ($<$ 250 μm), medium ($\ge$ 250 μm), and large ($\ge$ 400 μm) full-thickness macular holes (FTMH) treated with inverted internal limiting membrane (I-ILM) flap technique over a follow-up period of 12 months.

**Methods:**

Ninety-one eyes of 87 patients were enrolled in this retrospective study. BCVA and spectral-domain optical coherence tomography (SD-OCT) were conducted preoperatively as well as after 1, 3, 6, 9, and 12 months postoperatively. The defect length of the ELM and the EZ was measured using the caliper tool at each follow-up time point.

**Results:**

BCVA improved significantly in the group of small, medium, and large FTMH over the time of 12 months, whereby the improvement did not depend on FTMH size over 9 months. Only after 12 months, large FTMH showed significantly higher BCVA improvement compared to small and medium FTMH. The closure rate was 100% (91/91). The defect length of ELM and EZ reduced continuously over the period of 12 months. There was a significant correlation between defect length of ELM and EZ with postoperative BCVA.

**Conclusion:**

The I-ILM flap technique has very good morphological and functional outcomes in small, medium, and large FTMH over a long-time period, indicating that it can be considered as a treatment option in small and medium FTMH. The defect length of ELM and EZ is directly connected to postoperative BCVA.


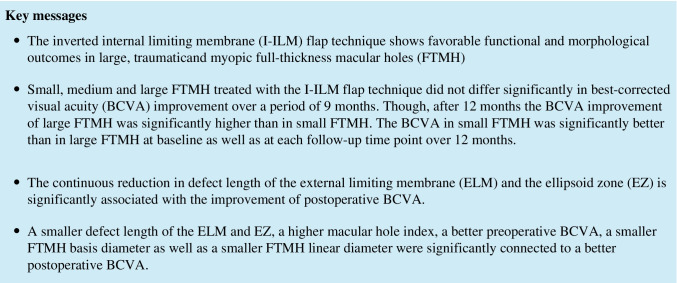


## Introduction

A full-thickness macular hole (FTMH) mostly occurs idiopathic and is defined as a macular lesion with the interruption of all retinal layers from the internal limiting membrane (ILM) to the retinal pigment epithelium (RPE). It is a common cause of significant visual impairment, metamorphopsia, and central visual field loss with a prevalence between 0.02 and 0.33%, while two thirds of the affected persons are female. Vitreoretinal traction has been considered as the key factor in the pathogenesis of idiopathic FTMH [1].

Since spectral-domain optical coherence tomography (SD-OCT) has been extensively applied to the diagnosis and prognosis of FTMH, various predictive preoperative and postoperative SD-OCT parameters have been discussed. A smaller preoperative minimal linear diameter of the FTMH in SD-OCT has been found to be associated with a better BCVA [2–7]. The International Vitreomacular Traction Study Group categorized FTMH according to the size of the minimal diameter of the FTMH on SD-OCT as follows: small ($\le$ 250 μm), medium (251–400 μm), and large ($>$ 400 μm) [2]. Still, the classification of FTMH and the consequences on decision for a therapeutic strategy have been discussed in various studies [8–11].

While pars plana vitrectomy (PPV) and intravitreal gas tamponade results in relatively high closure rates for small FTMH, additional ILM peeling improves closure rates and has become the mainstay treatment for FTMH with reported closure rates from 55 to 100% [12–15]. However, the success rate of hole closure is reduced in large FTMH [16–19]. In 2010, Michalewska et al. introduced the internal limiting membrane flap technique for the treatment of large FTMH [18]. Since then, several studies reported favorable anatomic and functional outcomes for large, traumatic, myopic, and chronic FTMH treated with this technique. Comparative studies and meta-analyses evaluating the I-ILM flap technique and the conventional ILM peeling have demonstrated better morphological and functional outcomes in FTMH treated with the I-ILM flap technique [20–29]. However, most of the studies included focused on large FTMH. Only few studies examined the functional and morphological outcomes of small and medium FTMH treated with I-ILM flap technique [9, 29, 30]. Two studies found significantly better postoperative BCVA in small and medium FTMH treated with the I-ILM flap technique compared to conventional ILM peeling as well as faster regeneration of retinal layers [9, 28]. Another study did not find any differences between the I-ILM flap technique and conventional ILM peeling regarding the postoperative BCVA and the integrity of retinal layers in small and medium FTMH [30]. The benefits of the I-ILM flap technique in FTMH of different sizes remain unclear and need to be further investigated.

The complete microstructural regeneration is another important prognostic factor for the functional outcome after FTMH surgery. Persisting photoreceptor layer discontinuity, in particular the external limiting membrane (ELM) and the ellipsoid zone (EZ), is associated with worse BCVA [31–38]. However, studies analyzing the quantitative longitudinal changes in the ELM and EZ as well as its relationship with changes in BCVA after FTMH surgery are necessary.

Our study aims to compare the functional and morphological outcomes of FTMH of different sizes after treatment with the ILM flap technique over a period of 12 months. To the best of our knowledge, we are the first study to analyze the microstructural defect length of ELM and EZ as well as the BCVA improvement after treatment with I-ILM flap technique in small, medium, and large FTMH.

## Methods

### Study design

This retrospective study was approved by the local ethics committee of the Technical University Munich and adhered to the tenets of the Declaration of Helsinki. All participants had given their written informed consent prior to surgery.

The consecutive records of patients who underwent surgery for FTMH repair using the I-ILM flap technique at the university hospital rechts der Isar of the Technical University Munich, Germany, between December 2009 and July 2020 were reviewed.^1^ Patients with coexisting ocular pathologies in the operated eye such as retinal vascular diseases (e.g., diabetic retinopathy, retinal vascular occlusion), age-related macular degeneration, glaucoma, history of previous retinal surgery, history of trauma, uveitis, high myopia (refractive error of more than − 6.00 diopters), or retinal detachment were excluded. Finally, 91 eyes of 87 consecutive patients were enrolled in this study.

Standard eye examinations were performed before surgery as well as 1, 3, 6, 9, and 12 months postoperatively, including BCVA, slit-lamp biomicroscopy, intraocular pressure measurements, and indirect ophthalmoscopy. Additional covariates collected were the patient’s age and gender, the duration of symptoms, lens status, and presence of an epiretinal membrane (ERM).

Spectral-domain optical coherence tomography (SD-OCT, Heidelberg, Spectralis) was conducted at baseline as well as 1, 3, 6, 9, and 12 months postoperatively. The minimum linear and base FTMH diameter and the defect lengths of the ELM and EZ were measured on their narrowest point parallel to the RPE using the manual caliper software tool. The macular hole index (MHI) (ratio of the macular hole height to the base diameter) was calculated for each patient [39].

The outer retinal layers of the ELM and EZ were defined as intact if a continuous hyperreflective line was displayed in SD-OCT. Any hyoreflective discontinuity of the ELM and EZ was classified as a disrupted layer. FTMH closure was confirmed via SD-OCT; a flat-open and elevated-open closure type configuration was considered as surgical failure. These classifications and measurement results are based on the agreement of two authors (M. M. and N. B.).

The International Vitreomacular Traction Study Group Classification System was used to divide the FTMH into three subgroups according to FTMH size depending on the minimal linear diameter as follows: small ($\le$ 250 μm), medium (251–400 μm), and large ($>$ 400 μm) [2].

The main outcome measure was the time course of changes in BCVA in the three subgroups. Secondary outcome measures were the FTMH closure and the changes in defect length of ELM and EZ.

### Surgical procedure

Standard three-port vitrectomy was performed using a 23-gauge system (DORC, Zuidland, The Netherlands) by a single surgeon (M. M.) in all patients. Phacoemulsification with intraocular lens (IOL) implantation was performed if a visually significant cataract was present. After core and peripheral vitrectomy, the ILM was stained with 0.025% Brilliant Blue G (Brilliant Peel, Fluoron, Germany). A potentially present ERM was differentiated from the ILM by its staining pattern and peeled consequently. In all cases, the I-ILM flap cover technique was performed creating a radial I-ILM flap (I-ILM flap rosette) to cover the FTMH [40, 41]. At the end of the surgery, 12% perfluoropropane (C3F8; Perfluoron, Alcon Laboratories, Fort Worth, TX, USA) was substituted in all cases. All patients were instructed to maintain a face-down position for 3 days after surgery. Dynamic intraoperative imaging with the microscope integrated iSD-OCT system Rescan 700 (Carl Zeiss Meditec AG, Oberkochen, Germany) was used to reassure a safe and controlled surgery with correct flap positioning at the end of surgery.

### Statistical analysis

For statistical analysis, the decimal visual acuity was converted to the logarithm of the minimum angle of resolution (LogMAR). SPSS (version 28.0; SPSS Inc., Chicago, IL, USA) was used for the statistical analyses. Continuous variables were reported as the mean ± standard deviation (SD) or median and range, whereas categorical variables were expressed as percentages and absolutes. Two-tailed standard *t* test, Chi-square test, or Mann–Whitney *U* tests were used for comparison of variables between two groups. Paired *t* tests were conducted to analyze postoperative changes in measured outcomes. Univariate variance models were conducted with postoperative BCVA as the dependent variable. *P* values < 0.05 were considered statistically significant. Post hoc statistical power analysis was performed using G*Power (version 3.1, 2014).

## Results

We included 91 eyes of 87 consecutive patients in the study. The mean age of the patients was 67 ± 7 years. Two thirds (65.9%, *n* = 60) of the patients were female. At baseline, 69 (75.8%) eyes were phakic, and 22 (24.2%) eyes were pseudophakic. Combined phacoemulsification and intraocular lens implantation with PPV were performed in 22 cases (24.2%). Lens status of patients during the follow-up was included as a possible confounder variable in our statistical analysis.

The mean minimal FTMH diameter at baseline was 395 μm (± 147 μm, range 105–863 μm). Divided into subgroups, 19 (20.9%) small (< 250 µm), 22 (24.2%) medium (≥ 250 µm), and 50 (54.9%) large FTMH (≥ 400 µm) were included. The mean FTMH base diameter was 809 μm (± 340 μm, range 217–2389 μm), and the mean central retinal height was 408 μm (± 78 μm, range 233–758 μm). The calculated mean MHI was 0.58 ± 0.27. The MHI showed a significant negative correlation to BCVA (LogMAR) at baseline as well as at 1, 3, 6, and 12 months postoperatively (*r* =  − 0.31, *r* =  − 0.49, r =  − 0.42, *r* =  − 0.32, *r* =  − 0.39, *p* < 0.05). A higher MHI was correlated with better postoperative BCVA values. Patients’ demographics and baseline characteristics are resumed in Table 1.

**Table 1:** Baseline characteristics of the patients (*n* = 87) and affected eyes (*n* = 91)

	In total	Small FTMH	Medium FTMH	Large FTMH
Age, years (mean $\pm$ SD; range)	67.0 ($\pm$ 7.0)	63.7 ($\pm 10.$ 7)	68.7 ($\pm$ 7.0)	66.7 ($\pm 6$.8)
Female gender, *n* (%)	60 (66%)	12 (63.2%)	13 (59.1%)	35 (70%)
Lens status, phakic	69 (75.8%)	14 (73.7%)	15 (68.2%)	40 (80.0%)
ERM, *n* (%)	44 (48.4%)	13 (68.4%)	12 (54.5%)	19 (38%)
Mean duration of symptoms, months (median), range	3.3, 0.3–27.6	2.7, 0.6–14.6	2.8, 0.3–10.8	4.9, 0.6–27.6
Mean FTMH minimal linear diameter in μm, range	395 (± 147), 105–863	203 (± 38.6), 105–241	319 (± 50.7), 251–400	502 (± 97.3), 401–863
Mean FTMH base diameter in μm, range	809 (± 340), 217–2389	512 (± 180), 217–936	702 (± 243), 384–1310	969 (± 331), 526–2389
Mean MHI	0.58 (± 0.27)	0.83 (± 0.43)	0.61 (± 0.17)	0.48 (± 0.14)
Preoperative BCVA (mean LogMAR ± SD), Snellen	0.83 (± 0.39), 20/125	0.56 (± 0.32), 20/80	0.81 (± 0.40), 20/125	0.93 (± 0.37), 20/160

*ERM*, epiretinal membrane; *FTMH*, full-thickness macular hole; *BCVA*, best-corrected visual acuity; *LogMAR*, logarithm of minimal angle of resolution; *SD*, standard deviation; *MHI*, macular hole index

FTMH closure was confirmed in 91 of 91 eyes within 3 months based on SD-OCT scans (closure rate 100%). We did not observe any case of flat-open or elevated-open FTMH. There were no adverse events during surgery or the follow-up period of 12 months in terms of a reopening. Postoperative data are shown in Table 2. An additional ERM peeling was performed in 44 (48.4%) eyes. We found no significant differences in baseline BCVA as well as BCVA and BCVA improvement 1, 3, 6, 9, and 12 months postoperatively between eyes with and without additional ERM peeling. An ERM was detected in 38% (*n* = 19/50) eyes with large FTMH, in 54,5% (*n* = 12/22) eyes with medium FTMH, and in 68.4% (*n* = 13/19) eyes with small FTMH. However, there was no significant correlation between FTMH size and ERM occurrence. The contingency coefficient only showed a tendency for a higher occurrence of ERM in small FTMH (*p* = 0.063). During the 12-month follow-up period, there was no observation of re-gliosis in any eye.

**Table 2:** Postoperative characteristics of the patients (*n* = 87) and affected eyes (*n* = 91)

	In total	Small FTMH	Medium FTMH	Large FTMH
FTMH closure, *n* (%)	91 (100%)	19 (100%)	22 (100%)	50 (100%)
BCVA 1 month postoperative (mean LogMAR ± SD), Snellen	0.47 ($\pm$ 0.29), 20/63	0.29 ($\pm$ 0.20), 20/40	0.40 ($\pm$ 0.15), 20/50	0.57 ($\pm$ 0.32), 20/80
BCVA 3 months postoperative (mean LogMAR ± SD), Snellen	0.42 ($\pm$ 0.25), 20/50	0.28 ($\pm$ 0.15), 20/40	0.44 ($\pm$ 0.31), 20/50	0.46 ($\pm$ 0.25), 20/63
BCVA 6 months postoperative (mean LogMAR ± SD), Snellen	0.37 ($\pm$ 0.30), 20/50	0.17 ($\pm$ 0.13), 20/32	0.35 ($\pm$ 0.20), 20/50	0.45 ($\pm$ 0.34), 20/63
BCVA 9 months postoperative (mean LogMAR ± SD), Snellen	0.32 ($\pm$ 0.28), 20/40	0.21 ($\pm$ 0.18), 20/32	0.27 ($\pm$ 0.24), 20/40	0.39 ($\pm$ 0.31), 20/50
BCVA 12 months postoperative (mean LogMAR ± SD), Snellen	0.33 ($\pm$ 0.20), 20/40	0.19 ($\pm$ 0.12), 20/32	0.36 ($\pm$ 0.23), 20/50	0.38 ($\pm$ 0.19), 20/50
BCVA improvement 1 month postoperatively (mean LogMAR ± SD)	− 0.33 ($\pm$ 0.34)	− 0.28 ($\pm$ 0.35)	− 0.34 ($\pm$ 0.24)	− 0.34 ($\pm$ 0.38)
BCVA improvement 3 months postoperatively (mean LogMAR ± SD)	− 0.42 ($\pm$ 0.36)	− 0.28 ($\pm$ 0.26)	− 0.41 ($\pm$ 0.37)	− 0.47 ($\pm$ 0.38)
BCVA improvement 6 months postoperatively (mean LogMAR ± SD)	− 0.48 ($\pm$ 0.40)	− 0.34 ($\pm$ 0.16)	− 0.49 ($\pm$ 0.30)	− 0.53 ($\pm$ 0.49)
BCVA improvement 9 months postoperatively (mean LogMAR ± SD)	− 0.52 ($\pm$ 0.44)	− 0.51 ($\pm$ 0.63)	− 0.54 ($\pm$ 0.38)	− 0.50 ($\pm$ 0.45)
BCVA improvement 12 months postoperatively (mean LogMAR ± SD)	− 0.50 ($\pm$ 0.35)	− 0.31 ($\pm$ 0.10)	− 0.39 ($\pm$ 0.21)	− 0.62 ($\pm$ 0.41)

*FTMH*, full-thickness macular hole; *BCVA*, best-corrected visual acuity; *logMAR*, logarithm of minimal angle of resolution; *SD*, standard deviation

**Fig. 1:**
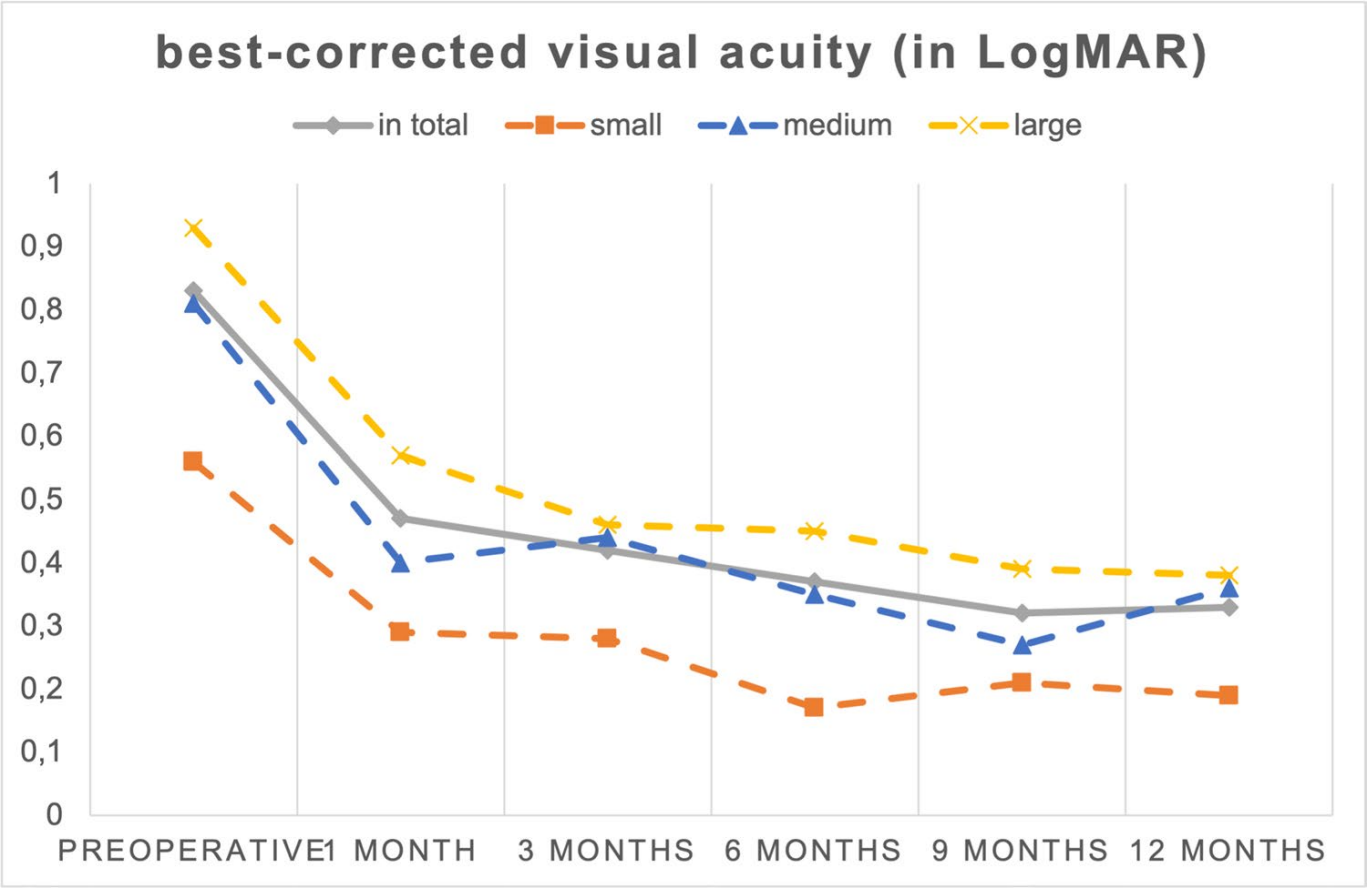
The mean BCVA improved from 0.83 ± 0.39 LogMAR preoperative to 0.47 ± 0.29, to 0.42 ± 0.25, to 0.37 ± 0.30, to 0.32 ± 0.28, and to 0.33 ± 0.20 LogMAR at 1, 3, 6, 9, and 12 months after surgery, respectively (*p* < 0.001). The mean BCVA improved from 0.56 ± 0.32 to 0.19 $\pm$ 0.12 LogMAR in small FTMH, from 0.81 ± 0.40 to 0.36 $\pm$ 0.23 LogMAR in medium FTMH, and from 0.93 ± 0.37 to 0.38 ± 0.19 LogMAR in large FTMH (*p* < 0.05)

**Fig. 2:**
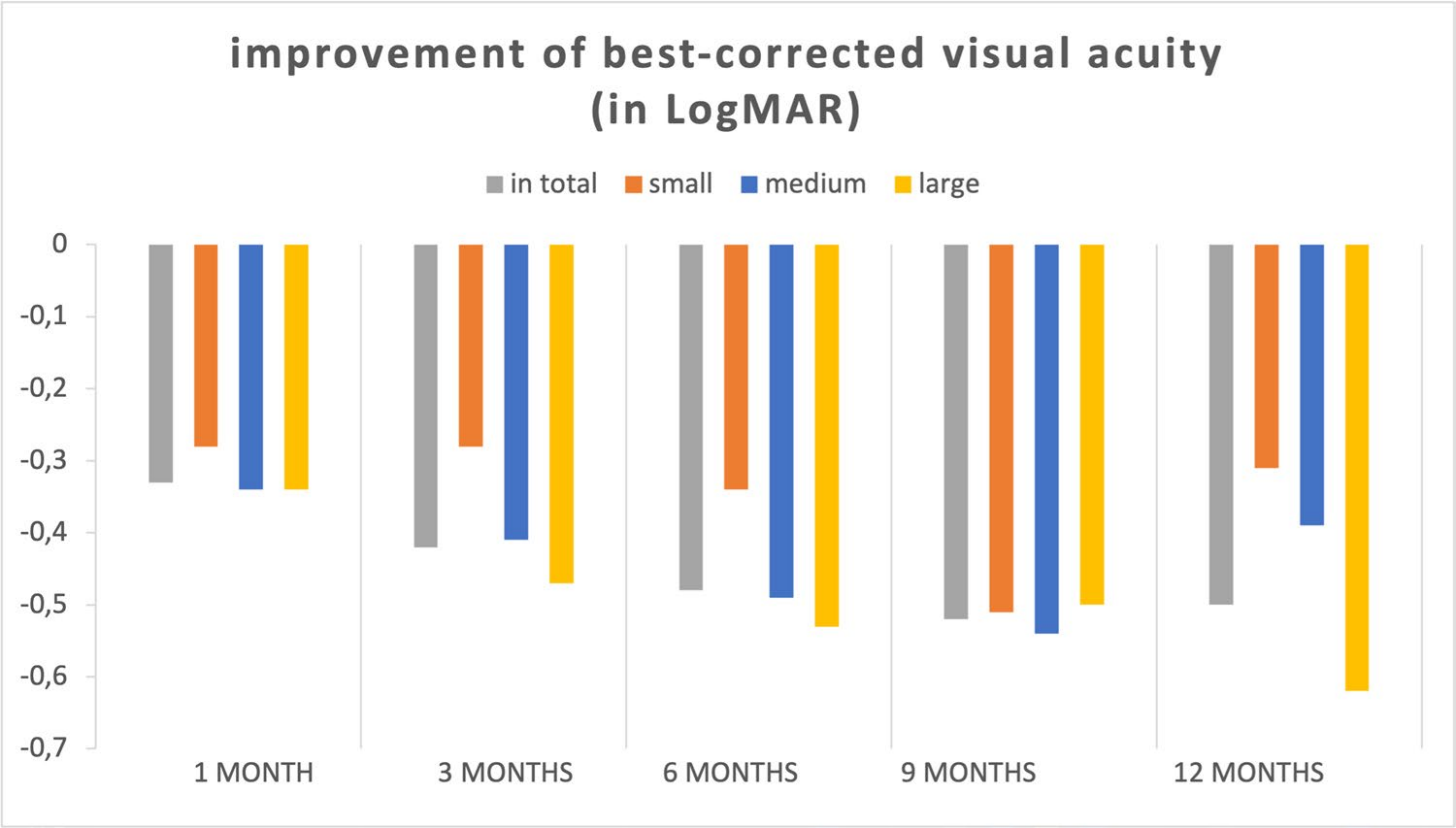
The BCVA improvement did not differ significantly between the three subgroups after 1, 3, 6, and 9 months postoperatively. Only after 12 months postoperatively, the BCVA improvement in large FTMH was significantly higher compared to small FTMH (*p* < 0.05). There were no significant differences in BCVA improvement between the group of small and medium FTMH as well as between the group of medium FTMH and large FTMH

The mean BCVA improved from 0.83 ± 0.39 LogMAR (Snellen’s equivalent 20/125) preoperative to 0.47 ± 0.29 (Snellen’s equivalent 20/63), 0.42 ± 0.25 (Snellen’s equivalent 20/50), 0.37 ± 0.30 (Snellen’s equivalent 20/50), 0.32 ± 0.28 (Snellen’s equivalent 20/40), and 0.33 ± 0.20 LogMAR (Snellen’s equivalent 20/40) at 1, 3, 6, 9, and 12 months after surgery, respectively (*p* < 0.001). Changes in preoperative and postoperative BCVA are displayed in Fig. 1. The mean improvement of BCVA equaled an improvement of approximately 5 Snellen lines. The postoperative BCVA improved in every patient compared to baseline BCVA. In small FTMH, the preoperative BCVA as well as at 1, 3, 6, 9, and 12 months postoperatively was significantly better than in large FTMH (*p* < 0.05). Also, the preoperative BCVA was significantly better in small FTMH compared to medium FTMH (*p* < 0.05) (Fig. 1, Table 2). There were no significant differences in BCVA between medium and large FTMH. BCVA improved significantly in each of the three subgroups over the period of 12 months, from 0.56 ± 0.32 LogMAR (Snellen’s equivalent 20/80) to 0.19 $\pm$ 0.12 LogMAR (Snellen’s equivalent 20/32) in small FTMH, 0.81 ± 0.40 LogMAR (Snellen’s equivalent 20/125) to 0.36 $\pm$ 0.23 (Snellen’s equivalent 20/50) in medium FTMH, and 0.93 ± 0.37 LogMAR (Snellen’s equivalent 20/160) to 0.38 ± 0.19 LogMAR (Snellen’s equivalent 20/50) in large FTMH (*p* < 0.05) (Fig. 2, Table 2). The BCVA improvement did not differ significantly between the three subgroups after 1, 3, 6, and 9 months postoperatively. Only after 12 months postoperatively, the BCVA improvement in large FTMH was significantly higher compared to small FTMH (*p* < 0.05). There were no significant differences in BCVA improvement between the group of small and medium FTMH as well as between the group of medium FTMH and large FTMH (Table 2).

**Fig. 3:**
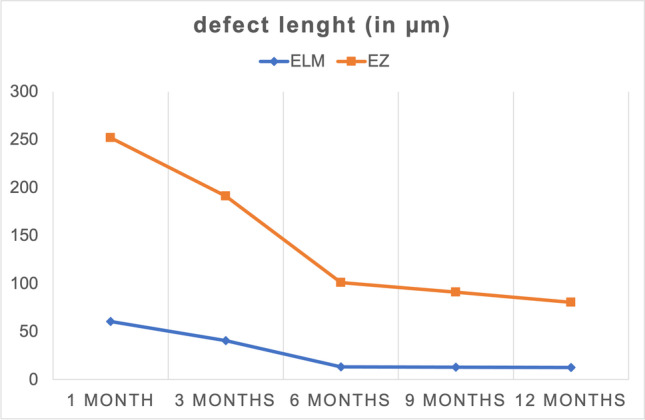
The mean reduction in defect length of ELM and EZ was continuously ongoing over a period of 12 months. Defect lengths of ELM were smaller as of EZ at all time points. The mean defect length of ELM at 1 month postoperatively was 60.5 μm (± 120 μm). It reduced significantly from 40.7 μm (± 84 μm) to 13.2 μm (± 57 μm), to 13 μm (± 50 μm), and to 0.12.8 μm (± 34 μm) at 3, 6, 9, and 12 months postoperatively, respectively (*p* < 0.05). The mean defect length of EZ at 1 month postoperatively was 252 μm (± 174 μm). It reduced significantly from 191 μm (± 167 μm), to 101 μm (± 102 μm), to 91 μm (± 121 μm),and to 80.6 μm (± 123 μm) at 3, 6, 9, and 12 months postoperatively, respectively (*p* < 0.05)

**Fig. 4:**
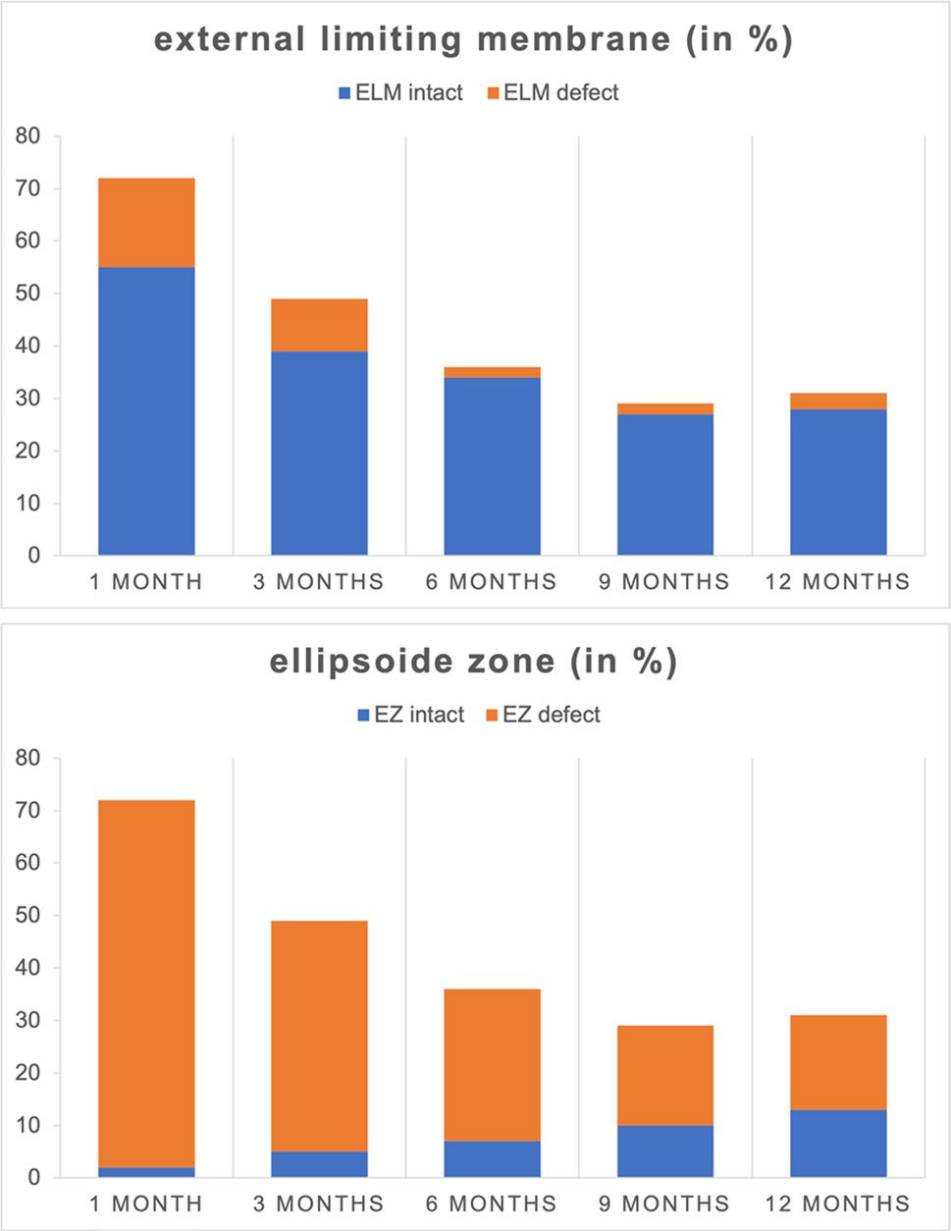
Regeneration of retinal layers in percent after 1, 3, 6, 9, and 12 months postoperative. SD-OCT showed complete restoration of foveal microstructures (ELM and EZ) at the end of the follow-up period in 41.9% (13/31) eyes. In 90.3% (28/31) an intact ELM and in 41.9% (13/31) an intact EZ were identified. Restoration of the ELM preceded restoration of the EZ in all cases. The integrity of the ELM and EZ was nominally graded as 0 if the layer was fully restored and continuous or as 1 if the layer was absent or partially restored but disrupted in SD-OCT

The mean defect length of ELM at 1 month postoperatively was 60.5 μm (± 120 μm). Divided into subgroups, it was 94.9 μm (± 132 μm) in large FTMH, 32.4 μm (± 120 μm) in medium FTMH, and 0 μm in small FTMH. The mean defect length of EZ at 1 month postoperatively was 252 μm (± 174 μm). Divided into subgroups, it was 288 μm (± 136 μm) in large FTMH, 257 μm (± 159 μm) in medium FTMH, and 150 μm (± 85.6 μm) in small FTMH. The defect lengths of the ELM and EZ reduced significantly over the course of time (*p* < 0.05) (Fig. 3, Table 3). A smaller size of FTMH led to a faster complete regeneration of the foveal microstructure. The ELM was completely intact in 28/31 (90.3%) eyes; the EZ was completely intact in 13/31 (41.9%) eyes 12 months postoperatively. Complete regeneration of the ELM preceded complete regeneration of the EZ in all cases. Therefore, the foveal microstructure (ELM and EZ) was fully restored in 13/31 (41.9%) eyes after 12 months postoperatively (Fig. 4, Table 3).

**Table 3:** Regeneration of ELM and EZ over 12 months postoperatively

Follow-up time point	ELM defect length (in μm, range)	EZ defect length (in μm, range)	ELM restored (in %, *n*)	EZ restored (in %, *n*)
1 month postoperatively	60.5 $\pm 120$(0–510)	252 $\pm 174$(0–868)	76.4% (55/72)	2.8% (2/72)
3 months postoperatively	40.7 $\pm 84$(0–312)	191 $\pm 167$(0–680)	79.6% (39/49)	10.2% (5/49)
6 months postoperatively	13.2 $\pm 57$(0–287)	101 $\pm 102$(0–420)	94.4% (34/36)	19.4% (7/36)
9 months postoperatively	13.0 $\pm 50$(0–226)	91.0 $\pm 121$(0–503)	93.1% (27/29)	34.5% (10/29)
12 months postoperatively	12.8 $\pm 34$(0–111)	80.6 $\pm 123$(0–485)	90.3% (28/31)	41.9% (13/31)

*ELM*, external limiting membrane; *EZ*, ellipsoid zone

The mean defect length of the ELM was significantly smaller than the defect length of the EZ at all time points postoperatively (*p* < 0.05). Larger defect lengths of the ELM and EZ correlated significantly with a poorer BCVA after 1,3, and 12 months postoperatively (ELM, *r* = 0.32, *r* = 0.35, *r* 0.39, *p* < 0.05; EZ, *r* = 0.45, *r* = 0.47, *r* 0.35, *p* < 0.05).

We validated prognostic factors concerning higher values of LogMAR and thus poorer BCVA of our previous study [40]. A larger base diameter and a larger minimal linear diameter of FTMH were connected to a worse BCVA (*r* = 0.52, *r* = 0.46, *p* < 0.001). A better baseline BCVA was associated with a higher postoperative BCVA (*r* = 0.48, *p* < 0.01). There were no significant correlations between gender, age, duration of symptoms, retinal thickness, and BCVA improvement in our study (*p* > 0.05).

The recorded median duration of symptoms was 3.3 months (range 0.3 to 27.6 months). As in our previous study [40], we found a statistically significant correlation between the duration of symptoms and the FTMH minimal diameter as well as the FTMH base diameter. A longer duration of symptoms related to a larger FTMH minimal diameter as well as a larger FTMH base diameter (*r* = 0.39, *r* = 0.32, *p* < 0.01). The duration of symptoms was significantly longer in the group of large FTMH than in the group of small or medium FTMH (*p* < 0.05).

The standard postoperative assessment in our clinic schedules visits after 1, 3, 6, 9, and 12 months postoperatively. However, not every patient meets all the scheduled appointments. Due to the retrospective design of our study, only 33 eyes (36.3%) completed the whole follow-up period. We analyzed the data for a possible bias concerning the patients who completed the follow-up in our clinic. We did not find a significant difference between patients who attended our clinic after 12 months postoperatively and those who did not. The composition of the follow-up collective did not differ significantly at the different time points concerning FTMH size, initial BCVA, age, and gender. The exact follow-up characteristics are displayed in Table 4.

**Table 4:** Follow-up characteristics of the patients (*n* = 87) and affected eyes (*n* = 91)

Follow-up time point	Eyes (*n*, %)	FTMH size large, medium, small (*n*, %)	Examination interval in months (mean $\pm$ SD, range)
1 month postoperatively	76 (83.5%)	42 (55%), 18 (24%), 16 (21%)	1.26 $\pm$ 0.4 (0.6–2.1)
3 months postoperatively	52 (57.1%)	31 (60%), 11 (21%), 10 (19%)	2.3 $\pm$ 0.8 (2.3–4.6)
6 months postoperatively	39 (42.9%)	22 (56%), 9 (23%), 8 (21%)	6.0 $\pm$ 0.8 (4.5–7.4)
9 months postoperatively	31 (34.1%)	16 (52%), 10 (32%), 5 (16%)	9.4 $\pm$ 1.2 (7.2–11.6)
12 months postoperatively	33 (36.3%)	19 (58%), 6 (18%), 8 (24%)	13.8 $\pm$ 2.8 (8.5–22.8)

*FTMH*, full-thickness macular hole; *SD*, standard deviation

## Discussion

Since the introduction of the I-ILM flap technique by Michalewska et al. in 2010, many modifications of this technique have been developed [18, 42–45]. In our study, we also modified the original I-ILM flap technique as we created a I-ILM flap rosette which covert the FTMH instead of a single I-ILM flap [40, 41]. The I-ILM flap technique and its modifications have been examined in various studies in the recent years showing favorable functional and morphological outcomes [20–29]. We confirmed FTMH closure in all eyes and found significant improvements in postoperative BCVA in general as well as in the three subgroups.

The application of the I-ILM flap technique has been described for large, traumatic, and myopic macular holes in the literature [20–29]. PPV and ILM peeling are the standard procedure for the treatment of small and medium FTMH [11]. Chou et al. examined FTMH smaller 400 µm treated with either I-ILM flap technique or conventional ILM peeling. They found favorable results in eyes treated with the I-ILM flap technique with less re-gliosis and faster regeneration of outer retinal layers as well as higher BVCA improvement in the first 6 months postoperatively [9]. The I-ILM flap technique should be considered as a treatment option for small and medium FTMH. In our study, we performed the I-ILM flap technique for medium and small FTMH as well as in large FTMH. To our knowledge, this is the first study to analyze differences of functional and morphological outcomes in different FTMH sizes. Comparing the BCVA improvement in eyes with small, medium, and large FTMH over the period of 12 months, we found no significant differences during the first 9 months. The preoperative BCVA as well as the BCVA after 1, 3, 6, 9, and 12 months was significantly better in the group of small FTMH compared to medium and large FTMH as well as it was better in the group of medium FTMH compared to large FTMH. After 12 months postoperatively the BCVA improvement was significantly higher in the group of large FTMH compared to the group of small or medium FTMH, indicating a longer ongoing process of BCVA improvement in the group of large FTMH and a higher BCVA improvement in total compared to preoperative BCVA. This can be explained by a “ceiling effect” as small FTMH have better preoperative BCVA and therefore cannot improve as much as large FTMH.

The leading hypothesis of FTMH closure mechanism after treatment with the I-ILM flap technique is the ILM flap to serve as a scaffold for Muller cells whose migration and proliferation promote the process of FTMH closure [46–48]. Morawski et al. established an in vitro model of the interaction between the ILM and the Muller cells which showed the ILM to be an optimal growth surface for Muller cells [46].

The microstructural regeneration of the outer retinal layers, especially of the ELM and the EZ, and their influence on the postoperative improvement of BCVA have been addressed more often over the last years. A complete regeneration of the ELM and EZ showed to be a prognostic factor for a better BCVA compared to eyes with remaining defects of the ELM and EZ [31–38]. To our knowledge, we are the first to assess the defect length of ELM and EZ over a period of 12 months and its direct connection to BCVA. Smaller defect lengths of the ELM and EZ were significantly associated to better postoperative BCVA. Therefore, we state that not only the full microstructural regeneration of the ELM and EZ predicts a better BCVA. Defect lengths of the respective retinal layers showed to be directly connected to postoperative BCVA in our study. The process of reintegration of the outer retinal layers is continuously ongoing over a period of 12 months or even longer. A fully regenerated ELM was identified to be essential for EZ regeneration [34, 40]. Our study validates this finding, as the ELM defects were smaller than the EZ defects at all time points, and a fully reintegrated ELM always preceded an intact EZ. Comparative research found a faster regeneration of the ELM and EZ in eyes treated with the I-ILM flap technique compared to treatment with conventional ILM peeling [9, 28, 49].

At 12 months postoperatively, 41.9% (13/31) eyes had a fully reintegrated ELM and EZ, while in 90.3% (28/31) an intact ELM and in 41.9% (13/31) an intact EZ were identified. Necessarily, patients should be educated about the long-ongoing process of microstructural reintegration and its connection to BCVA improvement. In our study, we focused on the defect length of ELM, and EZ while other studies have been performed on thickness of retinal layers. Lee et al. found an association between thickness of the inner retinal layer and better BCVA [50].

In our study, we confirmed prognostic factors concerning BCVA improvement [40]. A smaller defect length of ELM and EZ, a higher MHI, a better preoperative BCVA, a smaller FTMH basis diameter, and a smaller FTMH linear diameter were significantly connected to a better postoperative BCVA. A longer duration of symptoms correlated significantly with a higher linear diameter of FTMH. Therefore, we again pronounce the importance of an early diagnoses and treatment of FTMH [3, 10, 40]. This is emphasized by our finding that BCVA was significantly higher at each measure point for the group of small FTMH compared to medium and large FTMH.

Our study has several limitations. First, the number of eyes included reduced over the follow-up period of 12 months which led to missing data. This is explained by the retrospective design of our study as not every patient attended the recommended postoperative follow-up procedure. Further prospective studies with large sample sizes are necessary to validate our data. However, we did explicitly not exclude patients who were lost to follow-up as this may possess a significant selection bias. Second, we only addressed BCVA concerning the functional outcome. Different parameters like, e.g., retinal sensitivity should be further investigated. The strength over our study is the relatively large sample size and the standardized surgery procedure and analysis protocol over a period of 12 months. Comparative studies of the three subgroups treated with either conventional ILM peeling or with I-ILM flap technique should be conducted to further examine the functional and morphological benefits of the I-ILM flap technique not only for large but also for small and medium FTMH.

## Conclusion

In our study, the I-ILM flap technique showed very good functional and morphological outcomes in small, medium, and large FTMH over a period of 12 months. Each FTMH was closed postoperatively and BCVA improved significantly in all groups. The three subgroups only differed at the measure point of 12 months in BCVA improvement whereby the BCVA improvement of large FTMH was significantly higher than in small FTMH. Therefore, we state that small, medium, and large FTMH benefit equally from the I-ILM flap technique. It should be considered as treatment option in a small and medium sized FTMH, especially in a functional oculus unicus situation or an unfavorable FTMH configuration (e.g., large basis diameter) [39, 40]. Our results underline the importance of the regeneration of outer retinal layers for postoperative BCVA improvement. We showed that the reduction of defect length of ELM and EZ is continuously ongoing over a period of at least 12 months and that ELM regeneration precedes EZ regeneration. A higher defect length of ELM and EZ was directly connected to a poorer postoperative BCVA.

## Data Availability

All the data are available upon request.

## Footnotes

1 The I-ILM flap technique was presented by Michalewska et al. during the DOG conference in 2009.
